# Ability of FFR-CT to detect the absence of hemodynamically significant lesions in patients with high-risk NSTE-ACS admitted in the emergency department with chest pain, study design and rationale

**DOI:** 10.1016/j.ijcha.2020.100496

**Published:** 2020-03-05

**Authors:** David Meier, Ioannis Skalidis, Bernard De Bruyne, Salah Dine Qanadli, David Rotzinger, Eric Eeckhout, Carlos Collet, Olivier Muller, Stephane Fournier

**Affiliations:** a Department of Cardiology, Lausanne University Hospital, Lausanne, Switzerland; b Department of Radiology, Lausanne University Hospital, Lausanne, Switzerland; c Cardiovascular Center Aalst, Aalst, Belgium; d Division of Cardiology, Department of Advanced Biomedical Sciences, University of Naples Federico II, Italy

**Keywords:** Acute coronary syndrome, Coronary computed tomography, Fractional Flow Reserve, FFR-CT, AE, Adverse Event, ACS, Acute coronary syndrome, CT, Computed tomography, ECG, Electrocardiogram, ED, Emergency department, FFR, Fractional Flow Reserve, FFR-CT, FFR derived from coronary CT, Hs-Tn, High-sensitive troponins, MACE, Major adverse cardiac events, MI, Myocardial infraction, CMRI, Cardiac Magnetic resonance imaging, NSTE-ACS, Acute coronary syndromes without ST-segment elevation, NSTEMI, Non-ST-elevation myocardial infarction, PCI, Percutaneous Coronary Intervention, STEMI, ST-elevation myocardial infarction, URL, Upper Range Limit

## Abstract

**Background:**

In the era of High-sensitive troponin (hs-Tn), up to 50% of patients with a mild increase of hs-Tn will finally have a normal invasive coronary angiogram. Fractional Flow Reserve (FFR) derived from coronary computed tomographic angiography (FFR-CT) has never been used as a non-invasive tool for the diagnosis of coronary artery disease in patients with high-risk acute coronary syndrome without ST segment elevation (NSTE-ACS).

**Aims:**

The study aims to determine the role of coronary CT angiography and FFR-CT in the setting of high-risk NSTE-ACS.

**Methodology:**

We will conduct a prospective trial, enrolling 250 patients admitted with high-risk NSTE-ACS who will rapidly undergo a coronary CT angiography and then a coronary angiography with FFR measurements. Results of coronary CT, FFR-CT and coronary angiography (± FFR) will be compared.

**Potential significance:**

In conclusion, non-invasive identification of patients with high-risk NSTE-ACS who could avoid coronary angiography would reduce procedure related risks and medical costs.

## 1 Introduction

Patients admitted to the emergency department (ED) for chest pain are a top priority, the early diagnosis of a myocardial infarction (MI) being key for the prognosis of the patient. Unlike myocardial infarction with ST segment elevation (STEMI) where the diagnosis is usually straightforward after electrocardiogram (ECG) analysis, acute coronary syndrome without ST segment elevation (NSTE-ACS) patients represent a major diagnostic challenge. Indeed, clinical assessment and ECG alone are not sufficient to confirm or exclude diagnosis in most patients. Therefore, the addition of blood test such as troponin remains the cornerstone of an early diagnosis and subsequent adequate treatment especially regarding the decision to send the patient to the catheterization laboratory for a coronary angiography. The recent introduction of high-sensitive troponins (hs-Tn) has considerably improved diagnostic sensitivity of NSTE-AC. However, this comes at a cost as a high proportion of these patients will finally have a ‘normal’ invasive angiography (up to 50% among patients with an increase of troponins of ≤3x the upper range limit (URL) according to the recent fourth universal definition of myocardial infarction from the European guidelines [1]).

As troponin alone seems to be insufficient to correctly identify patients in real need of a coronary angiography, alternative diagnostic strategies are critically needed in order to avoid multiple unnecessary exams. In that sense, the identification on a non-invasive tool able to identify patients in whom coronary angiography will not allow to reveal a significant stenosis would be time, resources and cost saving.

Fractional Flow Reserve (FFR) derived from coronary computed tomography (CT) is a technique developed to calculate non-invasively the hemodynamic impact of a stenosis based in coronary geometries extracted from conventional CT images. The value derived from the blood flow simulation can be interpreted in the same way as the invasive FFR, which is the gold standard of coronary hemodynamics assessment. To date, no study has evaluated the benefit of FFR-CT in NSTE-ACS patients.

We designed this trial to assess whether FFR-CT is able to identify among high-risk NSTE-ACS patients, those without hemodynamically significant coronary stenosis by invasive FFR.

## 2 Methods

### 2.1 Study population

Detailed inclusion/exclusion criteria are reported in Table 1. Patients intended to be enrolled in the study must be ≥18 years old and have to present a rise and/or fall of high-sensitive cardiac troponins T (hs-cTnT, Roche, Switzerland) values measured on at least 2 timepoints with at least one value above the 99th percentile of the URL. They must also present with symptoms of ischemia and/or new or presumed new significant ST-segment–T wave changes on ECGs.

**Table 1:** Complete list of inclusion/exclusion criteria.

**Inclusion criteria:**
≥18 years old patients
Presenting a rise and/or fall of high-sensitive cardiac troponins T (hs-cTnT) values measured on at least 2 timepoints with at least one value above the 99th percentile of the URL and with at least one of the following:
Symptoms of ischemia
New or presumed new significant ST-segment–T wave (ST–T) changes
Informed consent signed
Presumed availability for follow-up up to 1 year

**Exclusion criteria:**
STEMI patients
Estimated glomerular filtration rate (eGFR) of <45 ml/min
Presence of very high-risk criteria:
Hemodynamic instability or cardiogenic shock
Recurrent or ongoing chest pain refractory to medical treatment
Life-threatening arrhythmias or cardiac arrest
Mechanical complications of MI
Acute heart failure
Recurrent dynamic ST-T wave changes, particularly with intermittent ST-elevation
Pregnant and breast-feeding women (women of child bearing potential must have a negative urine or blood pregnancy at screening)
Contra-indication to beta-blocker and/or nitroglycerin
Patients with diagnosis made using a troponin dosage other than hs-cTnT
Patients with prior coronary artery bypass grafting (CABG)
Patient with known severe heart failure (i.e Ejection fraction of left ventricle of <30%)
Patient incapable of judgement or under tutelage
Patient in emotional distress or other unstable psychical condition incompatible with informed consent signature

STEMI patient will not be included as well as, severe renal failure, pregnant and breast-feeding women, patients with diagnosis made using a troponin dosage other than hs-cTnT, patients with prior coronary artery bypass grafting (CABG), and patient with known severe heart failure. Importantly, patients presenting one or more very high-risk criteria according to the 2018 European guidelines on myocardial revascularization [2], and the 2014 American guidelines on NSTE-ACS [3] and thus requiring coronary angiography within 2 h will also be excluded. These criteria are the presence of hemodynamic instability or cardiogenic shock, recurrent or ongoing chest pain refractory to medical treatment, life-threatening arrhythmias or cardiac arrest, mechanical complications of MI, acute heart failure, and recurrent dynamic ST-T wave changes, particularly with intermittent ST-elevation

### 2.2 Study design and procedure

The present study is a single arm, double blinded, prospective trial. A summary of study procedure and schedule is provided in Table 2**.**

**Table 2:** Detailed study procedure.

	Screening		D0	D1	D3	1 month	6 months	12 months
**General evaluation**		**Inclusion**						
Informed consent (study-related)	x^1^							
Demographic data / Medical history (SoC)	x							
Eligibility criteria (study-related)	x^1^							
**Laboratory tests**								
Creatinine (SoC)	x		x		x			
Pregnancy test (study-related)	x							
**Clinical evaluations**								
History / Symptoms assessment (SoC)	x					x	x	x
Physical examination (SoC)	x		x			x	x	x
Electrocardiogram (SoC)	x		x	x	x	x	x	x
Bicycle test (SoC)								x
Echocardiogram (SoC)							x	
**Diagnosis procedures and measures**								
Coronary-CT (study-related)			x^2^					
Coronary angiography, Invasive FFR (SoC)			x^2^					
FFR-CT and FFRangio (study-related)			Between D0 and D14					
**Safety**								
AEs of interest: acute kidney failure, vascular complications, stroke (study-related)			x	x	x			
AEs of interest: MACEs (study-related)			x	x	x	x	x	x
SEs (study-related)			x	x	x	x		

1 Patients will be first pre-selected based on patient's file, and patients meeting main eligibility criteria will be informed about the study. After patient signed the informed consent, all eligibility criteria will be formally checked, and patients meeting all eligibility criteria will be included. ^2^Coronary CT should be done before coronary angiography, and both should be done within 24 h from patient initial admission to ED. SoC: standard of care, AEs: adverse events, SEs: serious events.

Patients admitted to hospital with high-risk NSTE-ACS and who accept to participate to the study will undergo a coronary CT angiography within 23 h after diagnosis in order to have time to proceed to coronary angiography within the recommended window time of 24 h. After realization of the CT, they will benefit from guidelines recommended treatment including a coronary angiography. According to internal and international guidelines, all efforts will be made to perform the coronary angiography within 24 h of ACS diagnosis [2], [3].

The coronary CT angiography procedure takes approximately 1–2 h (including logistical organization and patient transportation), and all efforts will be made not to delay the coronary angiography compared to patients who would not participate in the study. At the time of coronary angiography, patients will be blinded from the CT results as well as the direct treating physician and interventional cardiologist realizing the procedure, in order to avoid any influence in patients’ management. The procedure will be done using a 256-slice multi-detector CT (GE Healthcare Revolution CT, Chicago, Illinois, USA). Just before the examination, as routinely done for this non-invasive test to allow for optimal coronary vasodilation and visualization, oral Metoprolol (25–50 mg) will be administered if necessary to ensure a heart rate of 65 bpm or lower as well as one unique dose of sublingual nitroglycerine (400–800 mg) [4], [5], [6]. Parameters used for CT acquisition will be as following [7]: 100 kVp/550 mA for BMI < 25 (high definition mode), 100 kVp/550 mA for BMI included between 25 and 30, 120 kVp/600 mA for BMI > 30 (standard definition mode). Off line, an FFR-CT will be calculated with the data of the coronary CT angiography by blinded investigators in a central FFR-CT core laboratory (HeartFlow ®, Redwood City, CA 94063, USA). In parallel an FFRangio™ analysis will also be conducted on the invasive coronary angiography images [8]. Hemodynamically significant lesion will be defined as lesion with an FFR-CT value of ≤0.80.

During the invasive coronary angiography, FFR will be measured in all lesions with a visual diameter stenosis ≥30% using the PressureWire™ X Guidewire (Abbott, Chicago, Illinois, USA) with the following protocol: first, equalization of the pressure wire and the aortic pressure will be performed at the tip of the guide catheter prior to all measurements. Second, the pressure wire will then be advanced distal to the stenosis. Third, hyperemia will be obtained using intracoronary adenosine (150mcg for the right coronary artery and 200mcg for the left descending or the circumflex coronary arteries). Fourth, at the end of the procedure, the absence of a drift will be confirmed after a pull-back of the pressure to the same location as the initial equalization. Hemodynamically significant lesion will be defined as lesion with an FFR value of ≤0.80.

Of note, in case of coronary angiography showing no obstructive coronary disease, and thus not offering satisfactory explanation for the myocardial injury, patients will undergo cardiac magnetic resonance imaging (CMRI), to detect a potential alternative diagnosis (myocarditis, Takotsubo…). This later examination will however be left to treating physician’s discretion depending on the clinical context.

Both angiography-derived FFR (FFRangio™) and FFR derived from FFR-CT will be measured in the same position as invasive FFR and compared with this latter as gold standard.

### 2.3 Hypothesis and study endpoints

The hypothesis to be tested is that FFR-CT might have an interest in three aspects of the management of high-risk NSTE-ACS patients:

(1) FFR-CT discriminates those without any significant lesions defined by a negative invasive FFR value (>0.80) among all lesions with ≥30% diameter stenosis by invasive angiographic evaluation.
(2) FFR-CT discriminates those with a significant lesion (defined by a positive invasive FFR (≤0.80).
(3) When a clear culprit-lesion is evident (i.e. sub-occlusive vessel and ECG changes in the same territory), FFR-CT helps for the management of the non-culprit lesions (good correlation compared to invasive FFR).

Co-primary endpoints will be negative predictive value of coronary CT angiography and FFR-CT as compared to angiography with FFR and accuracy, sensitivity, specificity and positive predictive value of FFR-CT as compared to angiography with FFR.

Secondary endpoints will be (1) correlation of FFR-CT as compared to invasive FFR in lesions with at least a 30% stenosis, (2) negative predictive value of coronary CT as compared to angiography, (3) accuracy, sensibility, specificity and positive predictive value of coronary CT as compared to angiography, (4) accuracy, sensibility, specificity, negative predictive value and positive predictive value of FFRangio™ as compared to angiography (5) a priori treatment decision after angiography versus treatment decision according to invasive FFR, (6) a priori treatment decision after angiography versus treatment decision according to FFRangio™, (7) a priori treatment decision after angiography versus treatment decision according to FFR-CT, (8) natural history of deferred lesions according to invasive FFR, FFR-CT, CT and FFRangio™ in term of MACE

The following safety endpoints will be systematically recorded: Radiation dose received per patient (mSv), contrast media dose received per patient (ml), incidence of acute kidney failure defined as an increase of >1.5x the baseline creatinine value 3 days after the coronary-CT, incidence of vascular complications related to the coronary angiogram, incidence of stroke and incidence of major cardiac adverse events (MACE, composite outcome including cardiovascular death, myocardial infraction, and urgent revascularization).

### 2.4 Follow-up

**Fig. 1:**
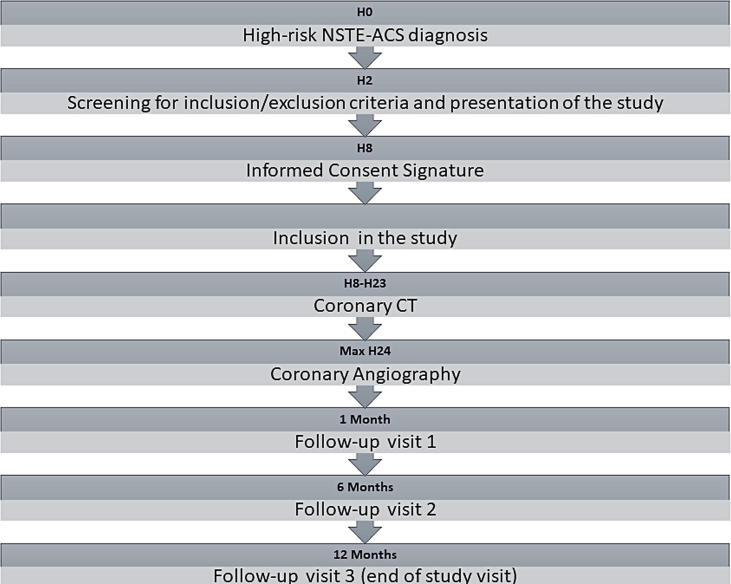
Flow-chart for patients included in the study.

Follow-up will be organized 1 month (±7 days), 6 months (±14 days) and 12 months (±14 days) after the acute coronary syndrome. These visits will include a detailed history, as well as physical examination and ECG. During the second visit, a transthoracic echocardiography will be performed, and a treadmill exercise stress test will be performed during the third visit. Study flow chart is presented in Fig. 1.

### 2.5 Data and statistical analysis

Statistical analysis will be carried out using SPSS 25.0 software (SPSS Inc., Chicago, Illinois) and Stata 14.3. Comparisons of characteristics of patients will be performed using Chi-squared tests for qualitative variables and Student’s t or Mann-Whitney tests as appropriate. Confidence intervals (CI) for proportions (sensitivity, specificity, accuracy) will be calculated using the Wilson Score method. With the last iteration of the FFR-CT software, the per-patient sensitivity and specificity were 86% (95% CI: 77% to 92%) and 79% (95% CI: 72% to 84%) [9]. In the recent VERDICT trial based on NSTEMI patients [10], 12% had 3 vessel disease, 23% 2 vessel disease (which included left main), 34%, 1 vessel disease, 30% nothing, Thus, out of 100 patients with 300 coronaries, we expect 106 (i.e. 36 + 46 + 34) vessels with a stenosis. Based on the FAMOUS NSTEMI trial [11], 60% of invasive FFR measurements of these stenoses were significant [12]. Thus, with a standard error of 0.05, and for a power of 80%, 204 patients will be required. However, some of the CT will not be suitable for analysis and some patients will have to be excluded. Therefore, we plan to include 250 patients. Of note, these number is also in line with the literature investigating the accuracy of FFR-CT versus FFR in patients with stable coronary artery disease [9], [13]. For the treatment decision based on the various methods, McNemar's test will be used.

### 2.6 Ethics

This project has been approved in May 2019 by the Medical Ethical Committee from canton de VAUD (CER-VD). The trial is also registered on ClinicalTrials.gov (NCT04052763).

This research project will be conducted in accordance with the protocol, the Declaration of Helsinki, the principles of Good Clinical Practice, the Human Research Act (HRA) and the Human Research Ordinance (HRO) as well as other locally relevant regulations

## 3 Discussion

### 3.1 Study population

Coronary CT as an adjunctive diagnosis tool as already been studied in the past, but in different sub-populations. In *a meta*-analysis of 9 studies (n = 1349 patients), CT presented an overall high negative predictive value to exclude ACS in patients presenting to the ED with chest pain and suspected of having an ACS [14]. The sensitivity (95% CI) of CT for NSTE-ACS diagnosis was 95% (88–100) and specificity (95% CI) was 87% (83–92), yielding a negative likelihood ratio (95% CI) of 0.06 (0–0.14) and positive likelihood ratio (95% CI) of 7.4 (4.8–10). However, in these studies, patients were in low to intermediate chest pain risk category with normal initial cardiac biomarkers and none had evidence of ischemia on initial ECGs and a NSTE-ACS was subsequently diagnosed in 10% of patients only.

Different randomized controlled trials have tested CT (n = 1869 patients) vs. usual care (n = 1397) in the triage of low- to intermediate-risk patients presenting with acute chest pain to ED without signs of ischemia on ECG and/or inconclusive cardiac troponins. At a follow-up of 1–6 months, there were no deaths, and a meta-analysis demonstrated comparable outcomes with the two approaches (i.e. no difference in the incidence of MI, post- discharge ED visits or rehospitalizations) and showed that Multiple Detector Computed Tomography was associated with a reduction in ED costs and length of stay. However, none of these studies used high-sensitivity cardiac troponin assays which is now the standard of care [15], [16], [17], [18].

FFR-CT is a rising technology and different studies – focusing on patient with stable coronary artery disease – have validated its good accuracy compared to invasive FFR. Per-patient sensitivity and specificity (95% CI) to identify myocardial ischemia (positive FFR) were 86 (77–92) % and 79 (72–84) % for FFR-CT versus 94 (86–97) % and 34 (27–41%) % for coronary CT in a study involving 254 patients [9]. In another study based on 252 patients [13], which did not achieve its prespecified primary outcome goal for the level of per-patient diagnostic accuracy (73% only), use of non-invasive FFR-CT among stable patients with suspected or known coronary artery disease was associated with improved diagnostic accuracy and discrimination compared to CT alone for the diagnosis of hemodynamically significant stenosis with FFR.

### 3.2 Study limitations

This study is not designed as a randomized trial, which obviously carries inherent limitations, associated with that type of design. However, it seems an appropriate design for two main reasons.

First, it is important to stress that, to date, no study has assessed the performance of FFR-CT in high-risk NSTE-ACS patients. Thus, it is unclear if performance of the technique is high enough to suppress the need for coronary angiogram in case of negative findings. In addition, a false-negative FFR-CT could have a potentially worse negative impact in an untreated NSTE-ACS who would be a higher risk of major cardiac events as compared to an untreated patient with stable angina. Second, even if the very large majority patients with an established myocardial infarction will have clear significant stenosis with easily identifiable culprit lesion (>60% with a >70% diameter stenosis and >30% with a > 90% diameter stenosis [11]), there are some patients that might also have an NSTE-ACS caused by plaque erosion only. Indeed, an optical coherence tomography study recently suggested that among an NSTE-ACS population of 492 patients undergoing percutaneous coronary intervention (PCI), there was ≈10% of cases with a culprit lesion caused by plaque erosion with a mean diameter stenosis of only 57% [19]. While we can hypothesize that in high-risk NSTE-ACS patients with critical stenoses, the sensitivity of coronary CT should be similar as the one observed in patients with stable coronary artery disease, preliminary data are important to investigate the ability of coronary CT to identify these instable eroded plaques (with modest diameter stenosis) in order to avoid discharging these patients.

Considering these two latter points, it seems premature to randomize patients to one approach or the other at that stage.

### 3.3 Clinical relevance

The questions that is sought to be answered by the present study seems relevant and important, as, cardiovascular diseases (and especially ischemic heart disease) remain the first cause of death in Western countries. Despite important improvement in diagnosis, therapeutic options and material, especially in patients with stable coronary artery disease and in patients with STEMI, the correct diagnosis of patients presenting with high-risk NSTE-ACS remains a daily challenge. Indeed, a significant number of patients with high-risk NSTE-ACS have a normal coronary angiogram which could have been avoided if another diagnostic procedure was available.

This especially relevant as they are exposed longer to a risk of bleedings while waiting for their coronary angiogram due to the administration of antithrombotic therapy and as, in a slight minority of cases, coronary angiography can lead to iatrogenic complications. Moreover, in many patients, coronary angiogram will identify multiples stenosis besides the culprit lesion and assessment of the hemodynamic significance of these stenoses might require multiples potential invasive FFR measurement. The latter are also associated with (rare) complications, require a lot of contrast media, radiation, and sometimes need to be done in more than one procedure.

To date, there is no simple non-invasive tool that might be applied on these high-risk patients as all the emerging techniques such as coronary CT or FFR-CT have only been studied in low-risk patients. Moreover, in a population of patients with recent STEMI and multivessel disease, FFR-CT has been shown to have only moderate accuracy in the evaluation of non-culprit lesions [20]. This fact might be explained by a smaller vessel volume and lower volume to mass ratio in the immediate post-STEMI period. It is still unknown if the same will apply in high-risk NSTE-ACS. In case of positive results of the present study, the next step would be to design a randomized trial assessing outcomes for patients assigned to one strategy or another, with a potential final perspective to use FFR-CT as a gatekeeper technique very early in the management of high-risk NSTE-ACS patients. This strategy would allow to classify these latter into 3 groups: those with non-coronary etiology, those with coronary etiology with an anatomy suitable for PCI, and those with complex multi-vessel disease needing CABG surgery. In the latter category, early identification might allow to avoid use of dual antiplatelet therapy and thus allowing for faster surgery with a lower bleeding risk.

Accordingly, the interest goes beyond its scientific aspects as the project might benefit directly to numerous patients, and as a public-health impact might expected with simpler management of this broad population. Finally, in times of growing health expenditures, this simple and non-invasive technique might contribute to cost control as it might spare numerous expensive procedures.

### 3.4 Project status and timeline

The project has begun to enroll patients in September 2019. Overall study duration should be 2.5 years as we expect to recruit patients over a 1.5-year period with a follow-up of 1 year for all patients. Indeed, the number of patients presenting with high-risk ACS in our institution is around 350 patients/year, but we anticipate that around 50% of patients will not be included (e.g. patients transferred from non PCI-capable hospitals with already too much time lost, patients not fulfilling all the inclusion/exclusion criteria, patients refusing to participate in the study, etc.).

## Funding

The present project is funded by a grant from the “Fondation Vaudoise de Cardiologie Interventionnelle”.

## Author contribution

All authors takes responsibility for all aspects of the reliability and freedom from bias of the data presented and their discussed interpretation.

## Declaration of Competing Interest

Dr Fournier reports an institutional consultancy for Bayer and Cathworks.

Dr. De Bruyne receives grant support from Abbott, Boston Scientific, Biotronik AG, and St Jude Medical and receives consulting fees on behalf of Dr De Bruyne from St. Jude Medical, Opsens, and Boston Scientific outside of the submitted work

Dr. De Bruyne is a shareholder for Siemens, GE, Bayer, Philips, HeartFlow, Edwards Life Sciences, and Ceyliad.

Dr. Collet reports receiving research grants from Biosensor, Heart Flow Inc. and Abbott Vascular; and consultancy fees from Heart Flow Inc, Abbott Vascular and Philips Volcano.
